# The Effect of Heavy Metal Concentration and Soil pH on the Abundance of Selected Microbial Groups Within ArcelorMittal Poland Steelworks in Cracow

**DOI:** 10.1007/s00128-012-0869-3

**Published:** 2012-11-08

**Authors:** Anna Lenart, Katarzyna Wolny-Koładka

**Affiliations:** Department of Microbiology, University of Agriculture in Cracow, Mickiewicza Ave. 24/28, 30-059 Kraków, Poland

**Keywords:** Heavy metals, pH, Soil, Soil microorganisms

## Abstract

The present study aimed to identify the effect of heavy metal concentration and soil pH on the abundance of the selected soil microorganisms within ArcelorMittal Poland steelworks, Cracow. The analysis included 20 soil samples, where the concentration of Fe, Zn, Cd, Pb, Ni, Cu, Mn, Cr and soil pH were evaluated together with the number of mesophilic bacteria, fungi, Actinomycetes and *Azotobacter* spp. In the majority of samples soil pH was alkaline. The limits of heavy metals exceeded in eight samples and in one sample, the concentration of Zn exceeded 31-fold. Chromium was the element which most significantly limited the number of bacteria and Actinomycetes.

Soil environment in industrial areas where the metallurgical plants are located, as well as in the agricultural areas surrounding these facilities is often heavily polluted with various xenobiotics, such as: policyclic aromatic hydrocarbons (Sofilič et al. 2008) and heavy metals, mainly: Cu, Mn, Zn, Cd and Pb (Rodella and Chiou 2009; Ettler et al. 2004). Increased heavy metal content negatively affects soil microbial population, which may have direct negative effect on soil fertility (Ahmad et al. 2005). Environmental pressure resulting from the contamination may reduce the biodiversity of microorganisms and disturb the ecological balance. However, there are reports stating that soil microorganisms may adapt to the increased, even toxic heavy metal and other xenobiotics’ concentration in soil (Kozdrój 1995) by developing various mechanisms to resist heavy metal contamination (Rathnayake et al. 2010).

Undoubtedly, soil microorganisms are essential for proper functioning of ecosystem and soil fertility. Chemical analyses often measure the particular amounts of contaminants but they do not reflect the environmental consequences resulting from their effect on key processes of soil metabolism. Biological methods can measure the actual impact of contaminants on soil organisms and they show the growth and activity inhibition under stress conditions (Šmejkalová et al. 2003) Given the above described relationships, the research was undertaken to assess the heavy metal contamination of soils in the area of ArcelorMittal Poland steelworks in Cracow and its effect on the abundance of the selected groups of soil microorganisms.

## Materials and Methods

Based on the analyses conducted previously (Lenart 2011), 20 soil sampling sites were selected in the area of ArcelorMittal Poland steelworks in Cracow. The exact location of the sampling sites are given in Table 1. The samples were collected in September 2011 into sterile polypropylene containers from the depth up to about 20 cm (ISO 1038). After being transported to the laboratory of the Department of Microbiology, University of Agriculture in Cracow, the samples were passed through a 2 mm sieve and analyzed by the serial dilutions method for the abundance of mesophilic bacteria (Trypticase Soy Agar, 48 h at 37ºC), fungi (Malt Extract Agar—MEA, 3 days at 28ºC), Actinomycetes (Pochon Agar, 5–7 days at 28ºC) and *Azotobacter* spp. (Ashby’s agar, 5 days at 26ºC). The number of colony forming units (CFU) of microorganisms was calculated per one gram of the soil dry weight. The analyses were performed in three replications and average values are presented. Additionally, soil moisture and pH were measured (ISO 1039; ISO 1146). The concentration of heavy metals (Fe, Zn, Cd, Pb, Ni, Cu, Mn and Cr) was determined using atomic absorption spectroscopy (AAS) following the procedure described by Akoto et al. (2008) and the evaluation of heavy metals’ availability was performed using Inductive Coupled Plasma Atomic Emission Spectrometry (ICP-AES) after extraction with CaCl_2_, as described by Galfati et al. (2011). The results were compared with Polish regulations for the maximum concentrations of heavy metals allowed in soils. Statistical analysis of the results was performed using Statistica software (StatSoft, USA), Pearson correlation (r) was applied to test relationships between the number of microorganisms and soil pH and heavy metal concentration at the level of significance equal to 0.05.

**Table 1:** Location and characteristics of 20 sampling sites within ArcelorMittal Poland steelworks in Cracow

Sample no.	Location	GPS coordinates	Soil pH	Soil moisture
1	Gate no. 2	N 50°04.958′ E 20°04.423′	8.6	14.92
2	Gate no. 3	N 50°05.286′ E 20°05.160′	8.0	12.37
3	Welded tube rolling mill	N 50°05.450′ E 20°05.735′	7.7	18.32
4	Cold rolling mill	N 50°05.050′ E 20°05.135′	8.24	14.85
5	Strip mill, below the pipeline	N 50°05.354′ E 20°05.887′	8.37	1.27
6	Hearth steel plant	N 50°04.674′ E 20°04.835′	8.65	23.40
7	Converter steel plant	N 50°04.216′ E 20°04.790′	8.20	24.87
8	Slabing rolling mill	N 50°03.956′ E 20°05.090′	8.59	12.24
9	Agglomerating plant 2	N 50°04.305′ E 20°06.047′	8.63	19.41
10	Cement plant	N 50°04.495′ E 20°07.018′	8.43	15.44
11	Coking plant	N 50°04.537′ E 20°06.042′	8.22	14.84
12	Biological treatment plant	N 50°05.098′ E 20°06.833′	9.20	14.10
13	Slag heap 1	N 50°05.000′ E 20°07.130′	9.40	10.61
14	Slag heap 2	N 50°04.450′ E 20°07.410′	9.10	16.89
15	Slag heap 3	N 50°03.639′ E 20°07.781′	8.78	5.28
16	Slag heap 4	N 50°03.657′ E 20°06.687′	11.40	23.60
17	Slag heap 5	N 50°03.896′ E 20°06.817′	8.60	13.05
18	Settling tank	N 50°03.681′ E 20°05.588′	8.20	11.09
19	Ash and sludge settling tank	N 50°03.450′ E 20°05.588′	8.46	39.45
20	Port channel	N 50°03.738′ E 20°05.687′	8.23	20.92

## Results and Discussion

Based on the data it was found that in 8 out of the 20 sites, the concentration of various heavy metals in the studied soil samples exceeded the limit values (Journal of Laws of the Republic of Poland 2002). The concentration of zinc exceeded the admissible values in samples: Gate no. 3 (the concentration of zinc in this sample exceeded 31-fold the limit value), Welded tube rolling mill (11-fold transgression) and Slag heap 2 (Table 2). Additionally the concentrations of cadmium, lead, copper and chromium exceeded the admissible values in 8 soils sampled from: Gate no. 3, Welded tube rolling mill, Coking plant, Slag heap 2, 3, 4 and 5, and Ash and sludge settling tank. The heavy metal concentrations varied significantly between the samples—e.g. the highest concentration of zinc recorded in the sample no. 2 was over 31,000 mg kg^−1^, while the lowest, recorded in the sample no. 8 was 37.46 mg kg^−1^. The mean values of the majority of heavy metals were higher than those reported by other Authors (Akoto et al. 2008; Kaszubkiewicz and Kawałko 2009), however the chromium concentration, even in the slag heaps, was lower than the one recorded by Huang et al. (2009) in samples collected from slag heaps of the steel alloy factory in China.

**Table 2:** Total heavy metal content in the analyzed soil samples within ArcelorMittal steelworks [mg kg^−1^]

	Metals							
Site no.	Fe	Zn	Cd	Pb	Ni	Cu	Mn	Cr
1	15,830	46.17	1.31	25.75	19.60	56.23	582.5	24.25
2	23,150	**31001.35**	**43.08**	**686.44**	20.60	225.84	383.65	43.20
3	24,140	**11658.20**	**16.07**	275.80	19.55	120.13	809.5	65.70
4	18,170	494.93	2.64	132.35	15.25	77.61	757	40.55
5	41,365	670.03	3.96	197.99	10.05	56.30	1378.5	25.80
6	27,225	322.31	3.73	168.59	20.40	81.00	933.5	67.75
7	28,270	186.10	1.30	110.44	12.70	70.39	886.5	26.65
8	18,200	37.46	0.09	8.63	12.90	29.11	634.5	17.65
9	69,700	262.64	1.47	129.55	15.90	99.74	2367.5	35.75
10	15,950	157.86	1.89	60.87	12.30	27.06	889.5	29.95
11	23,800	408.93	1.50	**1073.99**	35.25	**891.40**	761	116.50
12	32,405	279.11	1.46	50.06	16.75	71.11	3,715	29.25
13	41,220	824.5	2.65	104.4	48.35	61.45	5,685	670
14	46,485	**1600.5**	8.7	316.6	49.15	145.75	2,637	498.3
15	122,150	150.15	0.37	52.12	11.05	30.55	25,665	**862.00**
16	101,400	49.28	0.43	2.80	4.30	23.35	11,515	**590.00**
17	75,800	245.60	0.9	80.75	13.3	20.95	12,300	**629.5**
18	149,200	813.12	7.90	451.47	27.20	83.52	18,345	76.10
19	13,200	258.63	1.30	76.12	39.50	**610.37**	247.9	109.25
20	26,300	462.07	5.47	254.20	28.85	55.27	584	54.70
Mean	45698.00	2496.45	5.31	212.95	21.65	141.86	3728.35	200.64
SD	38511.59	7173.77	9.67	262.53	12.62	218.55	6215.68	274.48

Heavy metal contents which exceeded the admissible values (Regulation of the Minister of Environment of September 9th 2002) in bold letters

The water extractable and exchangeable, and organically bound fractions are considered as the most toxic fractions of Cd, Pb and Zn in soils in terms of the food chain input (Šmejkalová et al. 2003). The available forms of heavy metals varied between the analyzed sites and they were low as compared to the total heavy metal concentration (Table 3). Only in the case of Zn the concentrations were relatively high in three sampling sites.

**Table 3:** Content of available forms of heavy metals in the analyzed soil samples within ArcelorMittal steelworks [mg kg^−1^]

	Metals							
Site no.	Fe	Zn	Cd	Pb	Ni	Cu	Mn	Cr
1	0.105	0.395	0.015	0.115	0.075	0.130	0.125	0.030
2	**2.195**	**24.198**	**0.210**	0.135	0.055	0.070	0.370	0.025
3	1.705	**281.860**	0.095	**0.495**	0.085	0.185	0.595	0.040
4	0.255	**22.870**	0.075	0.115	0.070	0.155	0.640	0.0305
5	1.265	8.295	0.085	0.145	0.085	**0.425**	**1.055**	0.045
6	0.215	**22.470**	0.085	0.130	0.070	0.155	0.640	0.035
7	0.305	5.970	0.020	0.075	0.075	0.120	0.180	0.005
8	0.110	0.175	0.025	0.130	0.075	0.055	0.120	0.010
9	0.365	0.615	0.030	0.035	0.060	**0.290**	0.175	0.055
10	0.160	0.235	0.025	0.045	0.095	0.125	0.105	0.045
11	**2.205**	1.615	0.020	0.055	**0.120**	0.130	**1.054**	0.025
12	0.125	0.050	0.010	0.130	0.085	0.155	0.085	0.030
13	**7.875**	0.415	0.035	0.140	0.055	0.080	0.745	0.045
14	1.045	0.355	0.085	0.195	0.045	0.125	0.125	0.020
15	1.003	0.165	0.025	0.120	0.080	0.045	0.710	0.030
16	0.075	0.810	0.035	0.095	0.080	0.035	0.010	**0.685**
17	0.110	0.115	0.055	0.115	0.055	0.060	0.090	**0.105**
18	**3.020**	0.640	0.020	0.070	0.070	0.055	0.145	0.055
19	0.095	0.375	0.030	0.110	0.085	0.055	0.225	0.065
20	0.130	1.420	0.060	0.115	0.075	0.100	0.195	0.030
Mean	1.118	18.652	0.052	0.128	0.075	0.128	0.369	0.071
SD	1.819	62.497	0.046	0.094	0.017	0.093	0.333	0.146

The highest concentrations of metals in bold letters

In most samples, the soil pH fluctuated in the range from 7.7 to 9.40 and in one of the samples, (collected from the Slag heap 4) the pH was strongly alkaline: 11.40 (Table 1). This site is located in the old part of the metallurgical slag heap, which is currently being re-operated. The material at this site largely consists of slag, which may indirectly affect the distribution and abundance of microorganisms in this area (Huang et al. 2009), which was confirmed by our observations (Table 4).

**Table 4:** Abundance of the analyzed microorganisms in 20 soil samples within ArcelorMittal Poland steelworks [CFU g^−1^]

Analyzed microorganisms	Soil samples				
	1	2	3	4	5
Mesophilic bacteria	3,105,400	1,215,930	1,314,890	291,640	136,740
Fungi	1,070	5,700	1,600	1,590	14,450
Actinomycetes	68,700	1,480	15,700	7,200	99,500
*Azotobacter* spp.	1,656	0	0	0	0

	6	7	8	9	10
Mesophilic bacteria	195,390	2,583,520	1,853,160	726,720	352,410
Fungi	7,050	21,030	1,080	6,020	3,900
Actinomycetes	5,870	47,340	42,170	58,480	70,860
*Azotobacter* spp.	40	300	2,890	1,950	106

	11	12	13	14	15
Mesophilic bacteria	400,810	8,183,200	579,480	436,370	140,820
Fungi	12,172	12,360	1,290	57,905	4,040
Actinomycetes	20,260	44,380	27,370	17,650	500
*Azotobacter* spp.	0	130	0	570	0

	16	17	18	19	20
Mesophilic bacteria	0	104,040	2,250	40,740	10,435,950
Fungi	0	4,890	840	0	17,070
Actinomycetes	0	8,970	62	1,220	11,970
*Azotobacter* spp.	0	0	0	0	1,240

The number of mesophilic bacteria varied between the collected samples—from 10,435,950 in the sample collected at the Port channel to none in the sample Slag heap 4 (Table 4), which was characterized by the exceeded concentration of chromium and strongly alkaline pH (11.40). Moreover, none of the analyzed microbial groups were detected in this sample. This situation could have been caused mainly by very highly alkaline pH of the soil (Martyn and Skwaryło-Bednarz 2005), and by the increased chromium concentration (Megharaj et al. 2003). The highest abundance of fungi was observed in the sample Slag heap 2. It is the oldest metallurgical heap (mixture of production wastes without selective storage), also currently re-operated. These microorganisms were absent in two samples—the previously mentioned Slag heap 4 and Ash and sludge settling tank, where the increased copper concentration was detected. The number of Actinomycetes in the investigated samples was also varied and ranged from 99,500 CFU g^−1^ of soil in the sample Strip mill to 0 in the sample Slag heap 4. In general, this was low in comparison to the numbers recorded by Martyn and Skwaryło-Bednarz (2005) in non-contaminated soils and by Ahmad et al. (2005) in soils amended with various heavy metals. The analyzed soil samples differed also in terms of the abundance of *Azotobacter* spp. Populations of these bacteria in soils of neutral and alkaline pH rarely exceed several thousand cells per one gram of soil (Martyniuk and Martyniuk 2003). The results obtained in this study confirm this observation. The highest observed number of *Azotobacter* spp. was 2,890 CFU g^−1^ of soil in the sample with pH 8.59. These bacteria are also very sensitive to soil acidification and are rarely detected in acid soils (Martyniuk et al. 2007). It is impossible to verify this relationship in the studied area due to the fact that the majority of the analyzed samples was alkaline.

The analysis of correlation between the soil pH and the abundance of the analyzed microorganisms indicated weak relationship between these values (Table 5). Moreover, the statistical analysis revealed that the soil pH did not affect the number of fungi. Although the correlation was not statistically significant, it needs to be stressed that the presence of the tested microbial groups was not detected in the soil sample collected from the Slag heap 4, whose pH was 11.4. The statistical analysis of the effect of soil pH on the abundance of microorganisms may be hindered in this case due to the fact that the soil pH was generally alkaline, while the samples with acidic or neutral pH were not found among the studied ones.

**Table 5:** Correlation coefficients between microbial characteristics: total number and presence of the tested microorganisms and heavy metal content in soil and soil pH

	Fe	Zn	Cd	Pb	Ni	Cu	Mn	Cr	pH
Total heavy metal content									
Mesophilic bacteria	−0.27	−0.05	−0.02	−0.10	0.008	−0.17	−0.20	−0.31	−0.08
Fungi	−0.10	−0.06	0.05	0.19	0.42	0.04	−0.12	0.09	0.004
Actinomycetes	−0.33	−0.24	−0.26	−0.24	−0.27	−0.21	−0.32	−0.40	−0.12
*Azotobacter* spp.	−0.19	−0.18	−0.19	−0.25	−0.10	−0.20	−0.23	−0.30	−0.05
Available heavy metal content									
Mesophilic bacteria	−0.21	−0.05	−0.11	−0.19	0.07	−0.02	−0.29	−0.17	–
Fungi	−0.09	−0.14	0.15	0.06	−0.27	0.17	−0.07	−0.20	–
Actinomycetes	−0.11	−0.12	−0.24	−0.15	0.20	0.70	0.06	−0.24	–
*Azotobacter* spp.	−0.27	−0.16	−0.23	−0.14	−0.16	0.05	−0.38	−00.17	–

At the site gate no. 3, despite the recorded high concentrations of zinc, cadmium and lead (Zn—the allowable concentration exceeded 31-times; Cd-3- and Pb—the allowable concentration exceeded by 86 mg kg^−1^) a relatively high numbers of mesophilic bacteria and fungi were observed. The presence of Actinomycetes was also recorded at this site (1,480 CFU g^−1^), whereas the bacteria of the genus *Azotobacter* were not detected.

Based on the statistical analysis of correlation between the heavy metal content in soils and the abundance of the studied microbial groups, a weak negative correlation was found between the concentration of the majority of heavy metals and the number of *Azotobacter* spp. Only in the case of chromium this correlation was moderate. The same relationship was found in the case of mesophilic bacteria. Similarly for the fungi—the relationship between their numbers in soil samples and heavy metal content was very weak. Also the abundance of Actinomycetes was weakly correlated with the concentration of the heavy metals in soils. Only with regard to iron, manganese and chromium the moderate negative correlation was observed. These results are contrary to the findings of Šmejkalová et al. (2003) or Ahmad et al. (2005), who reported a significant decrease in CFU of most microbial groups with the increase of heavy metal concentration. Based on the described relationships it may be concluded that the analyzed microorganisms presented the potential resistance to toxic concentrations of heavy metals in the environment. The obtained results of statistical analysis suggest that chromium was the only element which most significantly limited the number of the microorganisms in soils. This metal presents strong toxic effect towards not only microorganisms, but also towards higher organisms (Shi et al. 2002).

In conclusion, the majority of soil samples were alkaline and in over one-third of them the heavy metal concentrations exceeded the allowable limits, however the observed differences in pH and the concentration of heavy metals only slightly affected the number of the analyzed microorganisms. Chromium was the element which most significantly limited the number of bacteria and Actinomycetes in soils of ArcelorMittal Poland steelworks in Cracow. The fact that the heavy pollution of some of the samples did not decrease the number of bacteria, Actinomycetes and fungi suggests that these microorganisms may present the resistance to increased or even toxic concentration of some heavy metals in soils and therefore may be used in future research on the bioremediation of contaminated areas.
